# Silymarin improved 6-OHDA-induced motor impairment in hemi-parkisonian rats: behavioral and molecular study

**DOI:** 10.1186/2008-2231-22-38

**Published:** 2014-04-11

**Authors:** Rasool Haddadi, Alireza Mohajjel Nayebi, Safar Farajniya, Shahla Eyvari Brooshghalan, Hamdolah Sharifi

**Affiliations:** 1 Student Research Committee, Tabriz University of Medical Sciences, Tabriz, Iran; 2 Drug Applied Research Center, Tabriz University of Medical Sciences, Tabriz, Iran; 3 Department of Pharmacology and Toxicology, Faculty of Pharmacy, Tabriz University of Medical Sciences, Tabriz, Iran; 4 Urmia University of Medical Science, Urmia, Iran

**Keywords:** Silymarin, Catalepsy, MDA, IL-1β, 6-OHDA, Rotarod, Rat

## Abstract

**Background:**

Neuroinflammation and oxidative stress has been shown to be associated with the development of Parkinson disease (PD). In the present study, we investigated the effect of intraperitoneal (i.p.) administration of silymarin, on 6-OHDA-induced motor-impairment, brain lipid per-oxidation and cerebrospinal fluid (CSF) levels of inflammatory cytokine in the rats.

**Results:**

The results showed that silymarin is able to improve motor coordination significantly (p < 0.001) in a dose dependent manner. There was a significant (p < 0.001) increase in MDA levels of 6-OHDA-lesioned rats whereas; in silymarin (100, 200 and 300 mg/kg, i.p. for 5 days) pre-treated hemi-parkinsonian rats MDA levels was decreased markedly (p < 0.001). Furthermore the CSF levels of IL-1β was decreased (p < 0.001) in silymarin (100, 200 and 300 mg/kg) pre-treated rats up to the range of normal non-parkinsonian animals.

**Conclusion:**

We found that pre-treatment with silymarin could improve 6-OHDA-induced motor imbalance by attenuating brain lipid per-oxidation as well as CSF level of IL-1β as a pro-inflammatory cytokine. We suggest a potential prophylactic effect for silymarin in PD. However, further clinical trial studies should be carried out to prove this hypothesis.

## Introduction

Parkinson’s disease (PD) is the second most common and progressive neurodegenerative disorder worldwide with a prevalence of approximately 1% in people over age 60. Clinically, the disease is characterized by resting tremor, rigidity, bradykinesia and postural instability [1-3]. Progression of these symptoms is secondary to the selective loss of dopaminergic (DA) neurons in the substantia nigra pars compacta (SNpc) [4]. The primary cause of PD is still unknown although aging appears to be a major risk factor. Indeed, mitochondrial impairment and elevated oxidative stress have been linked to the PD pathogenesis [5]. It is well agreed that chronic neuro-inflammation has part in the pathogenesis of the disease [6,7]. For the first time in 1988, McGeer et al. reported increase of activated microglia in the SNc of parkinsonian patients, which was showed the involving of neuroinflammation in PD [8]. Chronic neuro-inflammation causes to damage of neural cells through generation of reactive oxygen species (ROS). In the brain, activated microglia are a major origin of cytokines and oxidizing radicals which are produced subsequent to activation of intracellular peroxidases and oxidative processes [9]. ROS can activate pro-inflammatory pathways and subsequently cause to damage of vulnerable neurons. Several studies showed that both oxidative stress and neuro-inflammation play a role in the neurodegeneration of SNc observed in PD [10,11]. The dopaminergic neurons (DA-neurons) of SNc are highly vulnerable to oxidative stress due to the high oxygen demand of this brain region together with the low levels of antioxidant enzymes [12]. Previous studies have demonstrated increase of activated microglia in the SNc of parkinsonian patients [8]. It has been found that microglial activation result in the decrease of DA-neurons in patients suffering from PD [13,14]. Furthermore, the levels of proinflammatory cytokines such as tumor necrosis factor alpha (TNF-α), interleukin-1beta (IL-1*β*) and IL-6 that expressed by glial cells, increased markedly in the serum, brain and cerebrospinal fluid (CSF) of patients with PD [8,15,16]. To present, no effective therapies have been developed to treat PD; however, modulation of neuroinflammation is important to modify disease progression.

Silymarin (SM), is a polyphenolic flavonoid derived from the seeds and fruits of the milk thistle plant (*Silybum marianum*), routinely used to treat liver diseases and have antioxidative [17], anti-apoptotic [18], anti-inflammatory [19,20], and neuroprotective properties [21]. The anti-oxidative activity of SM is due to the scavenging of free radicals and activation of superoxide dismutase [22]. SM inhibits microglia activation and decreases inflammatory mediators; the mechanisms by which it protects dopaminergic neurons from lipopolysaccharide induced neurotoxicity [23]. Furthermore, several studies demonstrated protective effects of SM in several experimental models of neuronal injury, particularly in focal cerebral ischemia and cerebral ischemia–reperfusion-induced brain injury in rats [24,25]. However, less information is available about its effect on motor deficits associated with Parkinson disease. Therefore, the present study aims to investigate the effect of SM on 6-hydroxydopamine (6-OHDA)-induced motor imbalance and modification of cerebral levels of IL-1β and MDA as indicators of neuro-inflammation and oxidative damage.

## Material and methods

### Chemicals

All chemicals were purchased from Sigma Chemical Co. (USA). Solutions were made freshly on the days of experimentation by dissolving drugs in physiological saline (0.9% NaCl) except for silymarin which was dissolved in 50% polyethylene glycol (PEG). The drugs were injected intraperitoneally (i.p.) except for 6-hydroxydopamine (6-OHDA) which was injected into right substantia nigra pars compacta (SNc).

### Animals

Male Wistar rats (220 ± 20 g) were used in this study. The animals were given food and water ad libitum and were housed in standard polypropylene cages, four per cage at a ambient temperature of 25 ± 2°C under a 12-h light/12-h dark cycle. Animals were habituated to the testing conditions including being transferred to the experimental environment, handled, weighed, and trained on the test platform for 10 min 2 days before the behavioral investigations were conducted. The present study was carried out in accordance with the ethical guidelines for the Care and Use of Laboratory Animals of Tabriz University of Medical Sciences, Tabriz, Iran (National Institutes of Health Publication No. 85–23, revised 1985).

### Experimental protocol

In the beginning of study only the rats that showed normal walking on rotarod (700 ± 20 sec) were subjected to further experimentation. The healthy animals were randomized into 8 groups each consisting of eight rats. Rats in group 1 (control or intact) received no injection and were left untreated for the entire period of the experiment. Rats in group 2 (sham operated) were injected with saline containing 0.2% (w/v) ascorbic acid into SNc. Rats in group 3 received only 6-OHDA (8 μg/2 μl/rat; intra-SNc). Rats in group 4 pre-treated with vehicle PEG (i.p.) daily for 5 consecutive days and then were received intra-SNc injection of 6-OHDA in the same way as group 3. Rats in group 5 (positive control) pre-treated with i.p. administration of silymarin 200 mg/kg once daily (9 a.m.). Rats in groups 6 to 8 pre-treated with i.p. administration of silymarin (100, 200 and 300 mg/kg) once daily (9 a.m.) for 5 days and then were received intra-SNc injection of 6-OHDA in the same way as group 3 and then, after 3 weeks as recovery period, all animals were tested by rotarod.

At the end of experiments, the animals were anesthetized by i.p. injection of ketamine (80 mg/kg) and xylazine (10 mg/kg) and their cerebro-spinal fluid (CSF) were collected (as described below) and prepared for further analysis of IL-1β. Then animals were euthanized by an overdose of ether and the striatal tissue samples prepared for MDA assay.

### Surgical procedures

The animals were anesthetized by i.p. injection of ketamine (80 mg/kg) and xylazine (10 mg/kg). After the rats were deeply anaesthetized (loss of corneal and toe pad reflexes), they were fixed in a stereotaxic frame (Stoelting, Wood Lane, IL, USA) in the flat position. The scalp hairs were completely shaved, swabbed with povidone iodine 10% and a central incision made to reveal skull. The coordinates for this position were determined according to the rat brain in stereotaxic coordinates [26] anteroposterior from bregma (AP) = −5.0 mm, mediolateral from the midline (ML) = 2.1 mm and dorsoventral from the skull (DV) = −7.7 mm. Desipramine (25 mg/kg, i.p.) was injected 30 min before intra-nigral injection of 6-OHDA to avoid degeneration of noradrenergic neurons. Then 6-OHDA (8 μg/per rat in 2 μl saline with 0.2% ascorbic acid) was infused by infusion pump at the flow rate of 0.2 μl/min into the right substantia nigra. At the end of injection, cannula was kept for an additional 2 min and then slowly was withdrawn. Sham-operated animals were submitted to the same procedure except 2 μl vehicle of 6-OHDA (0.9% saline containing 0.2% (w/v) ascorbic acid) was infused into the SNc.

### Cannula verification

**Figure 1:**
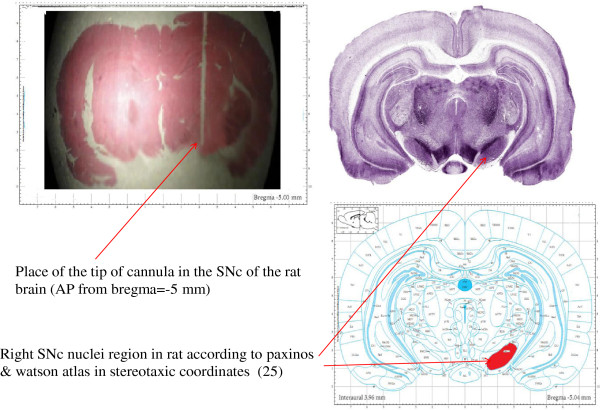
**Photomicrograph section of rat brain.** Placement of the cannula is shown according to the paxinos & Watson atlas in stereotaxic coordinates (25). Anteroposterior from bregma (AP) = −5.0 mm. Gomeri’s one-step Trichrom method (10 M).

For confirmation of placement of the cannula in the SNc of the brain, at the end of experiments all rats with guide cannula were euthanized by a high dose of ether and decapitated. The brains were removed and placed in a formaldehyde (10%) solution. After 1 week, the tissues were then embedded in paraffin. Then serial sections (3 μm) were cut with a microtome (Leitz, Germany), and as shown in (Figure 1), the placement of the tip of the cannula in the SNc, AP from bregma = −5.0 mm, was microscopically controlled (Figure 1). Data from rats with an incorrect placement of injecting cannula were excluded from the analysis.

### Rotarod assay test

**Figure 2:**
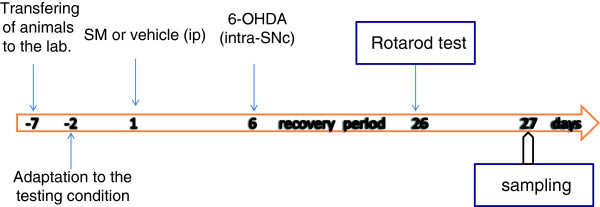
Schematic representation of the experimental procedure; see text for details.

Animals were transferred to the experimental room at least 1 h before the test in order to let them habituate to the test environment. Assessment of motor coordination and balance was done by a commercially available rat rotarod apparatus on the day of 21 after 6-OHDA injection (Figure 2). Rat was mounted on the rotarod (18 RPM) and the time latency to fall from the rod was automatically recorded. All observations were made between 9 AM and 4 PM by an observer who was blind to the entity of treatments. All rats were pre-trained for 2 days in order to reach a stable performance. The latency to fall from the apparatus was recorded. Motor balance was assessed three weeks after neurotoxin injection in four consecutive times, each lasting 720 s (with one hour interval). Values were expressed as retention time on the rotarod in the four test trails.

### CSF sampling

At the end of experiments, the anesthetized rats were mounted in a stereotaxic frame. The surface of the neck region were shaved and swabbed with ethanol (70%). The position of the animal’s head was sustained downward at almost 45°. A needle (scalp vein-23) which connected to a draw syringe was put horizontally and centrally into the cisterna magna for CSF collection with no incision at this region. The colorless CSF sample was slowly drawn into the syringe in a volume of 100 μl. The CSF samples were kept frozen at −70°C until assessment by enzyme-linked immunosorbent assay (ELISA) method.

### Malondialdehyde (MDA) assay

For MDA assessment, rats (n = 8) were euthanized, and selected brain regions (midbrain) were rapidly removed, cleaned, and immediately frozen in liquid nitrogen. Subsequently, tissue samples were weighed and homogenized (IKA Homogenizer, Staufen, Germany) in ice-cold buffer phosphate (50 mM, pH 6.0 at 4-8°C) and then were centrifuged at 10000 rpm for 20 min at 4°C. The supernatant was aliquot and stored at −80°C for further analysis. The MDA concentration in the supernatant was measured as described before [27]. Briefly, trichloroacetic acid and TBARS reagent were added to supernatant, then mixed and incubated at 100°C for 80 min. After cooling on ice, samples were centrifuged at 1000 × g for 20 min and the absorbance of the supernatant was read at 532 nm. TBARS results were expressed as MDA equivalents using tetraethoxypropane as standard. The protein content of the supernatant was measured using Bradford Protein Assay kit (Sigma Chemical, St. Louis, MO).

### IL-1β assay

The CSF level of IL-1β was determined by using commercial ELISA kits (Rat IL-1β kits, IBL, Hamburg, Germany) according to the manufacture’s instruction. Conditions were the same for all assays. Briefly, the frozen CSF samples were diluted, added into the wells and incubated at room temperature for 120 min on a microplate shaker. After washing, diluted Streptavidin-Horseradish peroxidase-conjugated anti-mouse IL-1β was reacted for 60 min at room temperature (on microplate shaker set at 200 rpm). After washing again, the wells were developed with tetramethyl benzidine (TMB) for 10 min and the optical densities were read at 450 nm with an ELISA reader. The concentration of the IL-1β was expressed as pg/ml of CSF.

### Statistical analysis

Data were expressed as the mean ± SEM, and were analyzed by one-way ANOVA in each experiment. In the case of significant variation (p < 0.05), the values were compared by Tukey test.

## Results

### The effect of intra-SNc-injection of 6-OHDA on motor- balance

**Figure 3:**
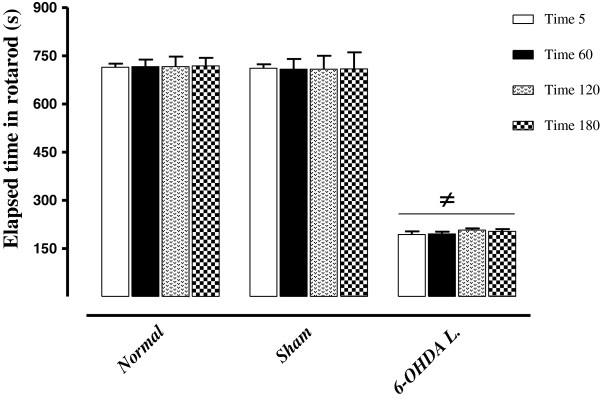
**The rotarod results of normal, sham-operated and 6-OHDA-lesioned (8 μg/2 μl/rat) rats.** Each bar represents the mean ± SEM of elapsed time on the rod (s); n = 8 rats for each group; ^≠^p < 0.001 as compared with normal and sham-operated groups. (L = .Lesioned).

The effect of intra-SNc injection of 6-OHDA on motor- coordination was evaluated by rotarod test. The duration of time to fall from rotating rod was evaluated in three groups of rats: normal, sham operated and 6-OHDA (8 μg/2 μl/rat)-lesioned rats. Drugs and vehicle were injected into the SNc through the implanted guide cannula. As shown in Figure 3, 6-OHDA was able to induce significant (p < 0.001) motor imbalance in comparison with both normal and sham-operated rats so that 6-OHDA- lesioned rats fail to maintain their equilibrium on rotarod and a significant decrease (367% in compare with normal group) in retention time on rotarod was observed in these animals (Figure 3).

### Effect of silymarin on 6-OHDA induced motor-incoordination

**Figure 4:**
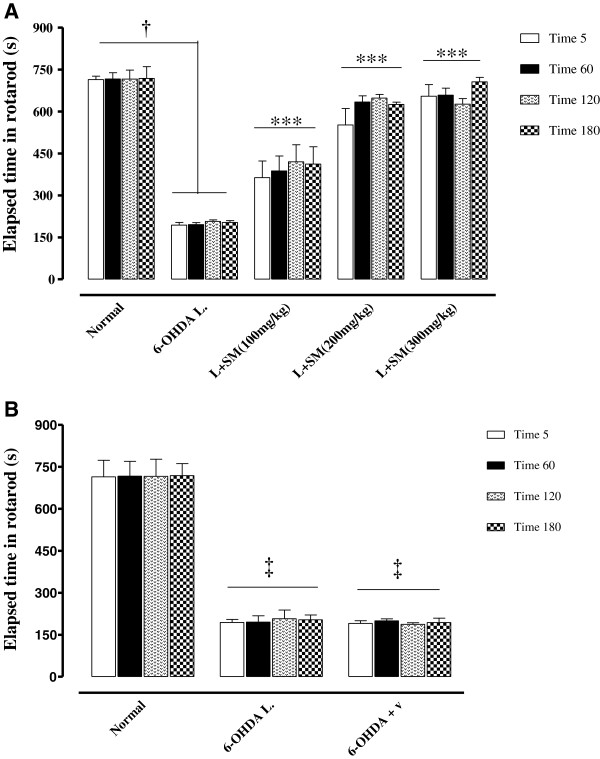
**The rotarod test results of 6-OHDA (8 μg/2 μl/rat)-lesioned rats that pre-treated with silymarin (100, 200 and 300 mg/kg, i.p. for 5 days) (A) and silymarin vehicle (B).** Each bar represents the mean ± SEM of elapsed time (s) on the rod; n = 8 rats for each group; ^†^p <0.001 between normal and 6-OHDA groups; ***p < 0.001 when compared with 6-OHDA lesioned rats; ^‡^p < 0.001 when compared with normal rats. (SM = Silymarin); (V = Vehicle of silymarin); (L = .Lesioned).

The effect of pre-treatment with silymarin (100, 200 and 300 mg/kg, i.p.) and its vehicle for 5 days, on 6-OHDA induced motor-incoordination was assessed 3 weeks after injection of 6-OHDA. In these groups motor balance was tested on 21 days after surgery for 4 repeated times (5, 60, 120 and 180 min). The results indicated that silymarin (in all 3 doses) significantly (p < 0.001) improved motor balance in 6-OHDA lesioned rats in a dose dependent manner (Figure 4A) so that the latency time to fall off increased (198%, 287% and 304%) in lesioned rats pre-treated with 100, 200 and 300 mg/kg of SM with respect to rats treated with vehicle, respectively. No alteration was observed on rotarod elapsed time in vehicle pre-treated rats (Figure 4B).

### Effects of 6-OHDA and silymarin on the brain level of MDA

**Figure 5:**
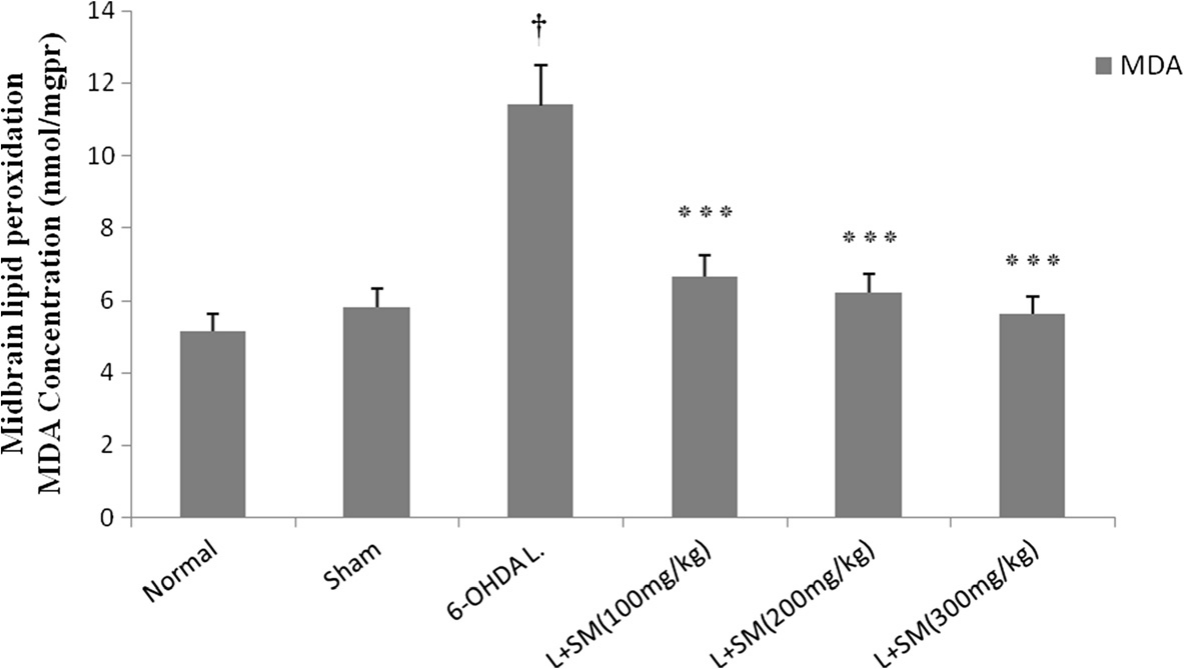
**The effect of i.p. administration of silymarin (SM) at the doses of 100, 200, and 300 mg/kg/day (for 5 days) on lipid peroxidation in midbrain as measured by MDA concentration.** Values are mean ± SEM (n = 8). ^†^P < 0.001 from respective normal value; ***P < 0.001 as compared with 6-OHDA injected group using one way ANOVA with Tukey post hoc test. (L = .Lesioned).

To investigate the possible involvement of MDA as a marker of the oxidative stress in PD we appraised the level of MDA in the ventral midbrain (brain region containing the SNc dopaminergic neurons). In the present study 6-OHDA injection given to the rats resulted in a significant (p < 0.001 vs. control) increase in lipid peroxidation in brain tissue, as measured by an increase in the level of MDA in the brain (Figure 5). The MDA level in the brain was found to reduce from 11.4 ± 1.1 nm/mg protein in 6-OHDA group to 6.62 ± 0.51, 6.25 ± 0.48 and 5.6 ± 0.63 (p < 0.001) nm/mg pr., in SM (100, 200, and 300 mg/kg/day) pre-treated groups, in a dose dependent manner, respectively (Figure 5).

### Effect of silymarin on the CSF level of IL-1β

**Figure 6:**
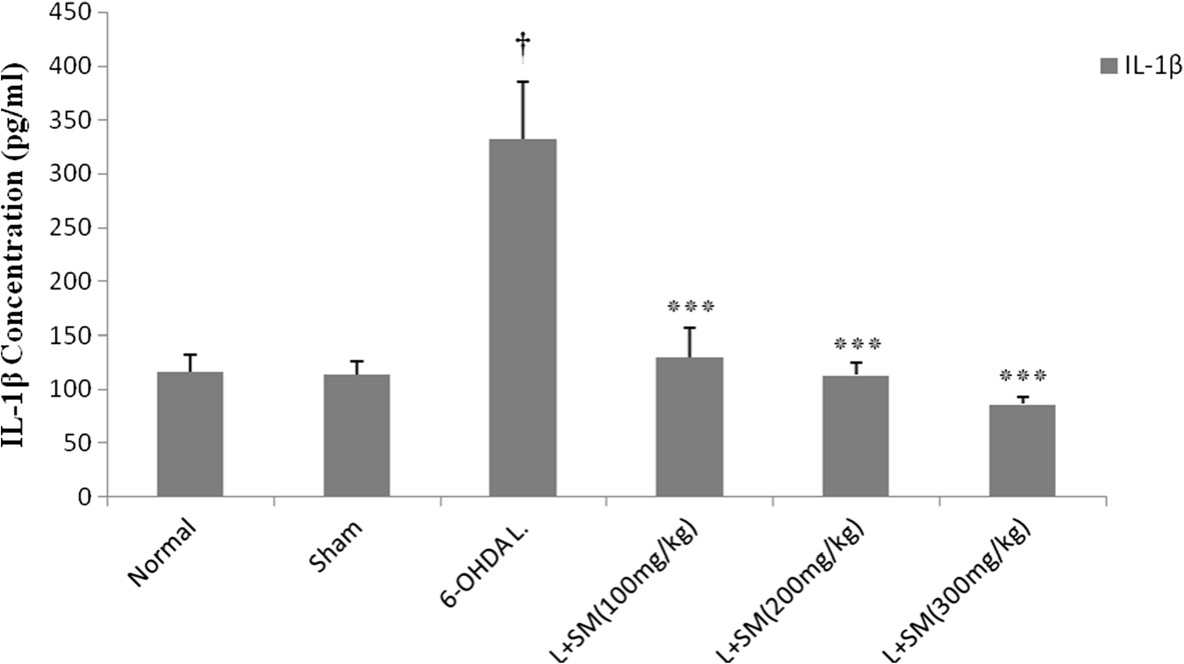
**The effect of i.p. administration of silymarin (SM) at the doses of 100, 200, and 300 mg/kg/day (for 5 days) on IL-1β concentration in CSF (Figure**6**).** Values are mean ± SEM (n = 8). ^†^P < 0.001 from respective normal value; ***P < 0.001 as compared with 6-OHDA injected group using one way ANOVA with Tukey post hoc test. (L = .Lesioned).

To further explore on the protection against 6-OHDA induced Parkinson disease by silymarin, the potential effect of silymarin on the level of IL-1β, which is a key pro-inflammatory cytokine released following microglia activation, was investigated in the present study. The results showed that 6-OHDA markedly increased the IL-1β level by 286.5% (P < 0.001 vs. control) (Figure 6). Pre-treatment with silymarin with all three doses (100, 200, and 300 mg/kg/day) significantly attenuated the level of IL-1β from 332.8 ± 52 pg/ml of CSF in the 6-OHDA group to 129.5 ± 26 (P < 0.001), 113.2 ± 11 (P < 0.001), and 86.16 ± 7 (P < 0.001) pg/ml respectively, in a dose-dependent manner (Figure 6).

## Discussion

We have previously reported that short-term administration of silymarin at induction of PD by 6-OHDA prevents from catalepsy and reduces myeloperoxidase activity and inflammatory cytokines [28]. In the present study it has been indicated that SM improves motor impairment following intra-SNc injection of 6-OHDA in rats and amends the neuroinflammation and oxidative stress factors.

Different beneficial effects of silymarin have been reported in several in vitro and in vivo studies. Recently, the beneficial effect of silymarin has been reported in an Alzheimer’s disease rat model [29]. However, less information is available about its effect on motor impairment in experimental models of PD. Behavioral assessment is a strong hallmark in evaluation of neuroprotection. Particularly, in rodents, motor-incoordination which is a reliable marker of the nigrostriatal neurodegeneration, is one symptoms of PD that can be created by unilateral intra-SNc injection of 6-OHDA and assessed by rotarod as a common standard motor-balance test [30]. In the 6-OHDA-induced PD model used in the present study, 6-OHDA caused a significant motor-imbalance, so that walking of rats on the rotating drum was lesser in 6-OHDA lesioned group compared to the control group. This is in accordance to previous studies reported that 6-OHDA (8-12 μg/2 μl/rat) induces motor-imbalance as an early symptom of PD by decreasing number of SNc neurons [30,31]. Indeed unilateral lesion of SNc compels a rat to put its weight abnormally on the both sides of its body for movement and equilibrium; hence this cause to motor disorders and motor asymmetry [31,32].

According to the results, silymarin (in all three doses) improved motor-incoordination induced by 6-OHDA. Baluchnejadmojarad et al. reported that silymarin attenuates the rotational behavior in 6-OHDA-lesioned rats and protects nigrostriatal neurons against 6-OHDA-induced neurodegenerative process [21]. 6-OHDA has pro-oxidant activity which causes to neurotoxicity. This toxin subsequent of auto-oxidation in the extracellular space, produce reactive oxygen species (ROS) [33]. Furthermore, it has been reported that 6-OHDA induce DA cells degeneration selectively by generation of free radicals and subsequently induction of oxidative stress and mitochondrial respiration dysfunction [34]. Additionally, oxidative stress result in microglia activation which leads to neurotoxicity of DA-neuron [14]. We suggest that the observed enhancement of motor balance in silymarin pre-treated hemi-parkinsonian rats in this study may be due to its possible neuroprotective effect. This effect may be exerted through counteracting oxidative stress process [35] maybe through regulating antioxidant defense system as well as inhibition of free radical generation [35].

To investigation of this issue, we also evaluated brain levels of MDA as lipid peroxidation marker in silymarin pre-treated hemi-parkinsonian rats. Malondialdehyde, a thiobarbiturate reactive substance, which formed as an end product of the peroxidation of lipids, served as an index of the intensity of oxidative stress. Our result showed a marked increase in midbrain lipid peroxidation as evidenced by elevation of MDA concentration in 6-OHDA-lesioned rats whereas, silymarin prevented dose-dependently from increase of MDA levels and restored it to the range of intact animals. According to these finding, we thought that silymarin attenuates the severity of oxidative stress through inhibition of lipid peroxidation. Also, it has been reported that the level of brain superoxide dismutase (SOD) and glutathione reductase (GR) as antioxidant enzymes decreased in parkinsonian rats and SM restored their levels in parkinsonian animals [21,36], which are totally in agreement with our findings.

Another key finding of the present study was that the reduction of brain MDA levels in silymarin pre-treated parkinsonian rats was associated with a significant decrease in pro-inflammatory cytokine. Our results showed that hemi-parkinsonian rats have a significant increase in CSF level of IL-1β, whereas its level was restored up to the normal range of intact animals by silymarin. This is in accordance with other previous studies which show that 6-OHDA- neurotoxicity induced by microglia activation and subsequent increase of TNF-α, IL-6 and IL-1β levels in both substantia nigra (SN) and striatum [8,37-39]. DA neurons in SNc are vulnerable to inflammatory affront as a major exciter of neurodegenerative disease because of high density of microglia in SNc [40]. Pro-inflammatory cytokines such as TNF-α, IL-6 and IL-1β are released by activated microglia in striatum and SN [41], which have an important role in neurotoxicity [42]. It was noted that SM reduced the levels of IL-6 and TNF-α as well as suppression of MPO activity in hemi-parkinsonian rats [28]. Furthermore silymarin inhibit nuclear factor kappa B (NF-kB) activation in DA neurons [23], and down-regulate cyclooxygenase-2 (COX-2) in brain [25], which could subsequently decrease release of IL-1β.

The anti-oxidant activity can be considered as a possible neuroprotective mechanism of silymarin in PD. It can be postulated that neuroprotective and anti-neuro-inflammatory effects of silymarin, in 6-OHDA induced hemi-parkinsonian rats, are almost due to reduction of pro-inflammatory cytokines and suppression of oxidative stress.

## Conclusion

In conclusion, we found that short-term pre-treatment with silymarin improved 6-OHDA-induced motor-imbalance and protected animals against neurotoxicity of 6-OHDA. Furthermore silymarin decreased CSF levels of IL-1β and decreased lipid peroxidation as evidenced by striatal level of MDA in 6-OHDA-lesioned rats. We suggest a possible protective role for SM against neuroinflammation and oxidative damages induced in PD. More clinical investigations should be done to prove its therapeutical application in PD.

## Competing interest

The authors declare that they have no competing interest.

## Authors’ contributions

RH involved in doing behavioral experiments and drafting. MNA the supervisor of the study participated and involved in concept, design, support of study, interpretation of data and final check of the draft. SF has made contribution in study as an advisor. SEB and HS involved in doing behavioral experiments. All authors read and approved the final manuscript.
